# A kurtosis-ESPRIT algorithm for RealTime stability assessment in droop controlled microgrids

**DOI:** 10.1038/s41598-024-84675-8

**Published:** 2025-01-13

**Authors:** Adham Osama, Abdallah F. El-Hamalawy, Mohammed E. Ammar, Amr M. AbdelAty, Hatem H. Zeineldin, Tarek H. M. EL-Fouly, Ehab F. El-Saadany

**Affiliations:** 1 Advanced Power and Energy Center (APEC), Electrical Engineering Department, Khalifa University, Abu Dhabi, UAE; 2 Electrical Power Engineering Department, Faculty of Engineering, Cairo University, Cairo, Egypt; 3 School of Engineering, Architecture, and Interior Design, Amity University, Dubai, UAE; 4 Engineering Mathematics and Physics Department, Faculty of Engineering, Fayoum University, Fayoum, Egypt

**Keywords:** Distributed generation, Droop control, ESPRIT technique, Kurtosis measure, Low-frequency oscillations, Microgrids, Small-signal stability, Electrical and electronic engineering, Energy grids and networks, Power distribution

## Abstract

Although detailed analytical models for droop-controlled microgrids are available, they are computationally complex and do not consider real-time variations in microgrid parameters and operating conditions. This paper proposes Kurtosis-Estimation of Signal Parameters via Rotational Invariance Technique (ESPRIT) to identify the dominant modes in droop-controlled inverter-based microgrids (IBMGs) using local real-time measurements. In the proposed approach, a short-duration small disturbance is applied to the selected DG’s active power droop gain, and then, the system’s dominant modes are estimated from its local measurements. Additionally, a kurtosis measure is proposed as a quick measure to assess the estimation signal’s characteristics and evaluate the presence and prominence of significant modes within the signal. The effectiveness of the developed approach is validated via MATLAB/SIMULINK simulations. Four case studies were conducted to verify the robustness of the proposed algorithm as follows: under different values of active power droop gains, several variations of lines’ X/R ratios, various levels of noise, and under large load changes and topological disturbances. Besides, a controller-in-the-loop (CIL) experiment was conducted using OPAL-RT to provide a real-time validation of the results. The modes obtained from the proposed algorithm are validated against the analytically derived modes and the estimation accuracy is compared to the recent methods: Prony, Matrix Pencil, and Subspace Identification techniques. Results show higher estimation accuracy for the proposed approach with a robust performance in noisy environments, across varying load conditions, and under different network configurations.

## Introduction

The surges in the penetration of renewable energy sources in modern power systems have been associated with a corresponding increase in distributed generation (DG) technologies^1^. The integration of DG systems allows for reduced dependency on fossil fuels which in turn minimizes the long-term costs associated with energy generation. Hence, microgrids have become crucial building blocks in the modern electric grid as they facilitate the operation of inverter-based distributed generation (IBDG)^2,3^. However, the operation and control of microgrids equipped with renewable energy sources (RESs) are challenging due to their associated intermittent behavior and low inertia compared to conventional synchronous generators^4^. Generally, the modern power system is continuously subject to changes. This is due to the stochastic variations of the loads and the dynamic nature of the power generation system^5,6^. Load variations lead to an oscillatory response in the system’s waveforms. Additionally, the reduced generation reserve margins, the rising demands, and the limitations of increasing transmission network capacity forced the grid operator to run the system closer to its technical limits^7^. Thus, these factors make the system more vulnerable to stability issues and frequent disturbances. These problems usually appear as poorly damped, low-frequency oscillations (LFO).

LFOs typically lie in the range of 0.2 to 3 Hz and are regarded as one of the major problems that can negatively impact the stability of the power system^8^. Generally, LFOs decay quickly and the system remains stable, but the stability of the power system will deteriorate if the LFOs are poorly damped which can lead to unstable system operation and even a major system blackout^9^. Thus, accurate identification of oscillation properties is of utmost importance for maintaining the safe operation of the power system and for initiating emergency control actions that preserve the system’s stability^10^. As a result, researchers’ interest in the dynamic monitoring of grid operations in real-time has grown extensively in the past two decades^11–13^. Low-frequency oscillatory modes can be identified using two approaches: model-based approach and measurement-based approach. The former is an off-line analysis that is based on the eigenvalue analysis of a system state-space matrix that is linearized around a certain operating point^14^. However, the power system has a dynamic nature with time-varying operating conditions, which makes it very difficult to construct an accurate model that accommodates all operating points. In addition, the complexity of large-scale power systems will make the construction of the system’s state-space matrix a very sophisticated process. Therefore, this approach is not suitable for high-order power systems^15^. The other approach for estimating oscillation parameters is based on measurements. Hence, the use of data-driven techniques has thrived with the expansion of wide area monitoring systems (WAMS) and phasor measurement units (PMU) in power systems^16,17^. These techniques employ advanced mathematical algorithms to identify key characteristics of power system oscillations.

Examples of recent measurement-based methods that have been used recently for the detection of LFO modes include the Kalman filter, Matrix Pencil^18^, Prony^19^, Subspace Identification (SID) technique^20^, and ESPRIT^21,22^. The Kalman filter is a recursive system identification technique, however, it has a problem of numerical instability^23^. A SID-based approach is used for the online assessment of the system’s stability, and it exhibits good performance in the presence of measurement noise^24,25^. However, SID techniques suffer from a high computational burden which makes them difficult to implement in a real-time environment. In^26^, the dynamic behavior of microgrids was investigated using Prony analysis and state-space modeling techniques. Prony analysis was introduced in^19,27^ as a modal identification technique to analyze the oscillatory behavior of the power system. However, Prony algorithm shows sensitivity to noise interfering with the measured signal, and although the improved versions of Prony can overcome this issue, they still suffer from a high computational burden. On the other hand, ESPRIT technique exploits the rotational shift invariance property of the signals to extract different signal parameters. Compared to Prony which uses the data samples directly to form the data matrix, ESPRIT uses the Hankel data matrix, which provides it with higher immunity toward noise^28^. In^29^, an estimate for power system harmonics and inter-harmonics was performed using an ESPRIT-based approach. Further, a detailed development of several robust signal processing algorithms including TLS-ESPRIT is provided in^30^. The numerical examples introduced in these studies verified the effectiveness of ESPRIT algorithm.

Based on the aforementioned review, it can be noticed that system identification techniques were widely applied in different power system applications, however, few research studies are available for the real-time monitoring and stability assessment in microgrids. In fact, dynamics in microgrids are considerably different from conventional power systems, especially for microgrids employing DGs with power electronic interfaces and renewable energy sources which are characterized by their intermittent nature. The dynamics in islanded microgrids are more rapid and nonlinear compared to dynamics in conventional systems which tend to be more stable and predictable. Thus, it is quite challenging to accurately capture the microgrid’s dominant modes under these fast-changing dynamics. This motivated the authors to propose a robust real-time stability assessment algorithm to continuously identify the system’s stability margin which can help network operators to design more robust controllers and take timely corrective actions, when necessary. The proposed approach relies on locally measured signals from the perturbed DG. Thus, it avoids the dependency on data communications, which are subject to reliability issues. Moreover, a clear understanding of the nature of the measurement signal is provided in this paper through the Kurtosis measure. This measure is generally utilized to detect outliers in the signal’s distribution which can indicate the presence of stronger and more dominant modes in the signal, especially in noisy environments. Kurtosis-based analysis can be combined with the ESPRIT algorithm in one tool to identify the dominant modes from the measured signal. Further, this tool can be combined with conventional decentralized microgrid controllers to enhance microgrid stability by updating the droop gains online. The contributions of this paper are summarized as follows:

- Proposing a robust stability assessment tool based on Kurtosis-ESPRIT algorithm for real-time monitoring of microgrids’ stability. To the best of the authors’ knowledge, this is the first study to demonstrate the effectiveness of ESPRIT for real-time stability assessment in droop-controlled inverter-based microgrids which hold a quite different and more challenging dynamic behavior compared to conventional power systems.
- Utilizing Kurtosis as a quick measure for describing the signal’s characteristics, indicating whether further detailed analysis, using ESPRIT, is likely to be more or less accurate.
- Assessing microgrids’ stability through local measurements only and thus, avoiding the use of PMUs and their associated communication channels.
- With its high estimation accuracy, ESPRIT analyzer demonstrates it can serve as an efficient alternative to the detailed small signal models when they are not available.
- Confirming the robustness of ESPRIT algorithm towards different levels of noise, across varying load conditions, and under different network configurations.
- Achieving higher estimation accuracy for the system’s dominant modes compared to the existing modal identification techniques discussed in the literature.

The organization of the rest of the paper is as follows: Section “Small-signal model-based analysis” presents the small-signal analysis of a generic islanded microgrid, which is used later to determine the accuracy of the proposed approach. In section “Kurtosis-ESPRIT-based stability assessment algorithm”, Kurtosis-ESPRIT algorithm is reviewed. Section “The proposed real-time stability assessment approach” illustrates the developed stability assessment tool. Case studies and simulation results are discussed and compared to the other existing algorithms in section “Simulation results”. A real-time validation for the proposed algorithm is introduced in section “Real-time validation of ESPRIT performance using OPAL-RT”. Finally, section “Conclusion” concludes the paper.

## Small-signal model-based analysis

**Fig. 1:**
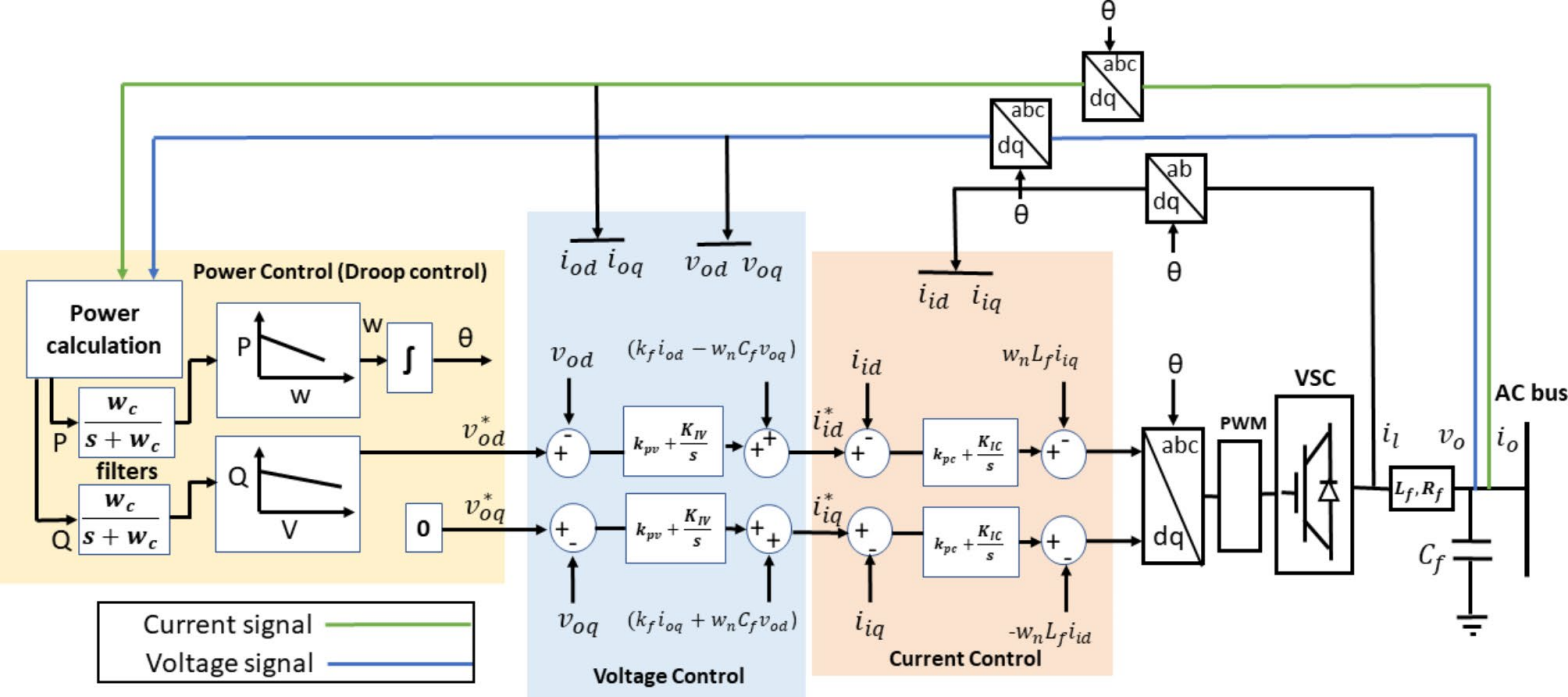
Block diagram of DG control loops.

The adopted small-signal model of an islanded microgrid using droop-based control was developed in^31^. The given modeling procedure can be applied for any number of DGs and various network configurations. Figure 1 shows the adopted control structure for the inverter-interfaced DGs. The voltage and current components in the dq frame are obtained, and the instantaneous active and reactive powers are calculated. These powers are filtered to produce the average values of active and reactive powers. In this control topology, the dq rotating frame is adopted for the derivation of the equations in the three control loops. The droop characteristics determine the microgrid’s frequency based on the active power, while the DG’s reference voltage is set based on the reactive power. The reference values of the current controller are set by the voltage controller. Both the current and voltage control loops are proportional-integral (PI)-based controllers that are utilized to provide efficient tracking for the signals. The final small-signal model of a DG is the resultant integration of the droop controller, current and voltage controllers, output filter, and coupling inductance. In turn, the small-signal model of an islanded microgrid considers the dynamics of the whole system including inverter dynamics in addition to network and load dynamics. The model of the $\:{i}^{th}$ DG can be represented as follows:

1 $$\:\left[\varDelta\:{x}_{INVi}\right]={A}_{invi}\left[\varDelta\:{x}_{INVi}\right]+{B}_{INVi}\left[\varDelta\:{V}_{bDQi}\right]+{B}_{i\omega\:com}[\varDelta\:{\omega\:}_{com}],$$

2 $$\:\left[\begin{array}{c}\varDelta\:{\omega\:}_{i}\\\:\varDelta\:{i}_{oDQi}\end{array}\right]=\left[\begin{array}{c}{C}_{INV\omega\:i}\\\:{C}_{INVci}\end{array}\right][\varDelta\:{x}_{INVi}]\:\left[\varDelta\:{x}_{INVi}\right]={[\varDelta\:{\delta\:}_{i}\:\:\varDelta\:{P}_{i}\:\:\varDelta\:{Q}_{i}\:\:\varDelta\:{ \phi }_{dqi}\:\:\varDelta\:{\gamma\:}_{dqi}\:\varDelta\:{i}_{ldqi}\:\:\varDelta\:{v}_{odqi}\:\:\varDelta\:{i}_{odqi}]}^{T},$$

where$\:\:\varDelta\:$, represents the small signal variation. The matrices A, B, and C determine the relationships between inverter state variables and input and output vectors. (*P*_*i*_, *Q*_*i*_) are the DG’s output active and reactive powers, and *δ*_*i*_ represents the angle between the local reference frame of each inverter and the global frame. (Φ_*dqi*_, $\:\gamma\:$_*dqi*_) are the errors integration of voltage and current expressed in their dq-frame components, respectively. The states (*i*_*ldqi*_, *i*_*odqi*_, *v*_*odqi*_) are the output currents and voltage in the dq rotating frame. ω_*com*_ is the common rotating reference frame frequency. The islanded microgrid’s small signal model includes the models of the inverters, network, and loads that constitute the microgrid. The model consists of (*h*) IBDGs and a network of (*m*) nodes that has (*r*) lines and supplies (*b*) loads. The microgrid’s small-signal model is represented as follows:

3 $$\:\dot{\left[\begin{array}{c}\varDelta\:{X}_{INV}\\\:\varDelta\:{i}_{line\:dq}\\\:\varDelta\:{i}_{load\:dq}\end{array}\right]}={A}_{mg}\:\left[\begin{array}{c}\varDelta\:{X}_{INV}\\\:\varDelta\:{i}_{line\:dq}\\\:\varDelta\:{i}_{load\:dq}\end{array}\right],$$

where *A*_*mg*_ is the microgrid’s state matrix. Δ*X*_*INV*_, Δ*i*_*line dq*_, *and* Δ*i*_*load dq*_ are the combined inverters’ state vector, the network lines’ current states, and the loads’ current states, respectively, which can be expressed as follows:

4 $$\:\varDelta\:{X}_{INV}={\left[\begin{array}{cc}\varDelta\:{x}_{INV\:1}&\:\varDelta\:{x}_{INV\:2}\end{array}\:\:\:\:\begin{array}{cc}\dots\:&\:\varDelta\:{x}_{IN{V}_{h}}\end{array}\right]}^{T}$$

5 $$\:\varDelta\:{i}_{line\:dq}=\:{\left[\begin{array}{cc}\varDelta\:{i}_{line\:dq\:1}&\:\varDelta\:{i}_{line\:dq\:2}\:\end{array}\:\:\:\:\begin{array}{cc}\dots\:&\:\varDelta\:{i}_{line\:d{q}_{r}}\end{array}\right]}^{T}$$

6 $$\:\varDelta\:{i}_{load\:dq}={\left[\begin{array}{cc}\varDelta\:{i}_{load\:dq\:1}&\:\varDelta\:{i}_{load\:dq\:2}\:\end{array}\:\:\:\:\begin{array}{cc}\dots\:&\:\varDelta\:{i}_{load\:d{q}_{\text{b}}}\end{array}\right]}^{T}$$

The stability and damping of the system can be evaluated using the microgrid’s state matrix *A*_*mg*_, which allows the identification of its eigenvalues. If all eigenvalues have negative real parts, the system is stable. Conversely, if any eigenvalue of the state matrix has a positive real part, the system is then unstable. The detailed modeling and analysis of the proposed inverter-based microgrid are given in^31^. Further, participation factor analysis was performed in this work to determine the contribution of different state variables to the overall system response. As presented in^32^, the participation of a certain state variable can be described as the measure of involvement of the *k*^*t*h^ state variable on the *i*_*th*_ mode and it can be expressed as follows:

7 $$\:{P}_{ki}=\frac{\left|{V}_{ki}{W}_{ki}\right|}{\sum\:_{i=1}^{M}\left|{V}_{ki}{W}_{ki}\right|}\:,$$

where *V*_*ki*_ and *W*_*ki*_ are the left and right eigenvectors of the state matrix *A*_*mg*_. The magnitude of *V*_*ki*_ represents a measure of involvement of the *i*^*th*^ mode *Z*_*i*_*(t)* in the *K*^*th*^ state variable *X*_*k*_*(t)*. The magnitude of *W*_*ki*_ represents the influence of the *K*^*th*^ state variable *X*_*k*_*(t)* in the *i*^*th*^ mode *Z*_*i*_*(t)*.

## Kurtosis-ESPRIT-based stability assessment algorithm

### Kurtosis statistical analysis

Kurtosis is a statistical measure used to describe the characteristics of a dataset. In^33^, it was defined as a descriptive statistic that measures how data is dispersed between a distribution’s tails and its center. Kurtosis was also characterized as “a measure of data peakedness or flatness relative to a Gaussian distribution” as mentioned in^34^. The normal distribution curve of a dataset is always depicted as a bell-shaped curve with a Kurtosis equal to three (K = 3) where most of the distribution of data is concentrated around the mean^35^. Large Kurtosis (K > 3) is normally characterized by thickly concentrated tails that hold extreme values or outliers, while low Kurtosis (K < 3) indicates a distribution with shorter tails and a more uniform distribution of data that lacks significant extremes. In this regard, a low Kurtosis value close to 3 for the measured data distribution can indicate that ESPRIT algorithm will have more difficulty identifying the dominant modes from the measured signal especially if the signal includes white Gaussian noise which has a kurtosis of three^36^, then the presence of these modes might be masked by the noise content. Thus, a low Kurtosis value in the presence of noise doesn’t necessarily imply the absence of dominant modes, rather, it may indicate that noise is overshadowing the signal features. Mathematically, the Kurtosis of a dataset can be calculated as follows:

8 $$\:K=\frac{E{\left(\widehat{x}-\stackrel{-}{x}\right)}^{4}}{{\left(E{\left(\widehat{x}-\stackrel{-}{x}\right)}^{2}\right)}^{2}}$$

where $\:E$ is the expectation operator, $\:\widehat{x}$ is the measured data vector, and $\:\stackrel{-}{x}$ is the mean of the data vector $\:\widehat{x}$.

### ESPRIT analysis background

ESPRIT is one of the signal processing techniques used to decompose complex signals in the form of the sum of exponentially damped sinusoidal signals. ESPRIT demonstrated high reliability and good estimation accuracy in terms of analyzing signals, especially in the presence of noise^28^. Several implementation methodologies were adopted for ESPRIT algorithm including the autocorrelation matrix of the signal^23,37^. In this paper, the measurement data vector was converted into a Hankel matrix instead of using the data samples directly or forming an autocorrelation matrix. This step provides the algorithm with higher immunity towards noise and allows more accurate estimations for the system’s dominant modes^28^. In addition, the measurement data were filtered through low-pass filters that remove high-frequency noise and allow only lower-frequency components to pass, besides DGs’ output filters that provide further cleaning for the signal.

The preprocessed low-frequency oscillation (LFO) signal is represented as follows:

9 $$\:\widehat{x}\left(n\right)=x\left(n\right)+r\left(n\right)\:=\sum\:_{i=1}^{p}{A}_{i}{\text{e}}^{{{\upalpha\:}}_{\text{i}}{T}_{s}{n\:}}\text{cos}\left(2\pi\:{f}_{i}{T}_{s}n+{\theta\:}_{i}\right)+r\left(n\right),$$

where *n* = 0,* 1*,* 2*,* ….*,* J-1*,* J* is the signal sampling points, *x(n)* is the original low-frequency signal, *T*_s_ is the sampling period, *r(n)* is the residual noise, $\:\widehat{x}$*(n)* is the measured LFO signal, *P* is the number of signal modes or the frequency components in the signal, and *α*_*i*_, *f*_*i*_, *θ*_*i*_, *and A*_*i*_ are the damping factor, frequency, initial phase, and amplitude of the $\:{i}^{th}\:$mode respectively. Equation (9) can be rewritten as follows:

10 $$\:\widehat{x}\left(n\right)=x\left(n\right)+r\left(n\right)=\sum\:_{i=1}^{2p}{C}_{i}{Z}_{i}^{n}+r\left(n\right),$$

where *Z*_*i*_ is the signal pole and it is equal to $\:{\text{e}}^{{({\upalpha\:}}_{\text{i}}+j2\pi\:{f}_{i}){T}_{S}}$, *C*_*i*_ represents $\:{\frac{1}{2}{A}_{i}e}^{j{\theta\:}_{i}}$ .

In this study, *x(n)* represents the batch of measurements taken after the perturbation removal until the oscillations are damped. ESPRIT algorithm follows the following steps to estimate the frequency components and their corresponding damping factors:

#### Step 1

The data vector of the selected measurement of a length *J* is arranged as a Hankel matrix of order *M*. *L* is a parameter representing the number of Hankel matrix rows and it should be given first where *L > 2P*,* M > 2P*, and *J = L + M-1*. The Hankel matrix is constructed from the signal *x(n)* in the following manner.

11 $$\:H=\left[\begin{array}{cccc}x\left(0\right)&\:x\left(1\right)&\:.\:&\:x(M-1)\\\:x\left(1\right)&\:x\left(2\right)&\:.&\:x\left(M\right)\\\:.&\:.&\:.&\:.\\\:.&\:.&\:.&\:.\\\:x(J-M)&\:x(J-M+1)&\:.\:&\:x(J-1)\end{array}\right]$$

The selection of the proper data vector size plays a critical role in achieving a clearer separation between signal and noise subspaces. In general, larger data vectors enhance resolution and improve estimation accuracy. Accordingly, in this study, the data size is defined such that the measurement data are taken immediately after the removal of perturbations and continue until the system reaches its steady state. This strategy ensures a balance between the accuracy of the algorithm’s estimations and the reduction in computational time.

#### Step 2

The singular value decomposition (SVD) is used to decompose the Hankel matrix in step 1. It divides the matrix into a signal subspace (*S*) and a noise subspace (*N*) as follows.

12 $$\:H=U\sum\:{V}^{H}=\left[{U}_{S}\:\:{U}_{N}\right]\:\left[\begin{array}{cc}\sum\:\:S&\:0\\\:0&\:\sum\:N\end{array}\right]\left[\begin{array}{c}{V}_{S}^{H}\\\:{V}_{N}^{H}\end{array}\right],$$

where *U* and *V* are two unitary matrices containing signal and noise subspaces. *∑* is a diagonal matrix that comprises all the singular values of *H*. The model order can be determined from the diagonal matrix. In this work, the fifth value in this matrix has a noticeable decline compared to the first four values. Thus, the model order was chosen to be four.

#### Step 3

The signal subspace is then divided into two shifted matrices and from the signal subspace *U*_*s*_ in step 2 by removing the last and first row of.

13 $$\:{U}_{1}=\left\{{u}_{1},{u}_{2},\:\dots\:,{u}_{M-1}\right\}$$

$$\:{U}_{2}=\left\{{u}_{2},{u}_{3},\:\dots\:,{u}_{M}\right\},$$

where *U*_*1*_ε* C*^*(M-1)×2P*^, and *U*_*2*_ε* C*^*(M-1)×2P*^ are the two shifted subspaces.

#### Step 4

The matrix *V*_*new*_ can be constructed as follows.

14 $$\:{V}_{new}=\left[\begin{array}{cc}{U}_{1}&\:{U}_{2}\end{array}\right]$$

Then applying SVD to the matrix *V*_*new*_ to get: $\:{V}_{new}=\stackrel{-}{U}\sum\:^{-}{\stackrel{-}{V}}^{H}$ where $\:\stackrel{-}{U}\in\:{C}^{\left(M-1\right)\times\:\left(M-1\right)}$, $\:\sum\:^{-}\in\:{C}^{\left(M-1\right)\times\:\left(4P\right)}$, $\:\stackrel{-}{V}\in\:{C}^{\left(4P\right)\times\:\left(4P\right)}$, and $\:\stackrel{-}{V}$ is divided into four matrices *2P×2P*

15 $$\:\stackrel{-}{V}=\left[\begin{array}{cc}\stackrel{-}{{V}_{11}}&\:\stackrel{-}{{V}_{12}}\\\:\stackrel{-}{{V}_{21}}&\:\stackrel{-}{{V}_{22}}\end{array}\right]$$

#### Step 5

Using the shift-invariance property, the rotation matrix Ψ is obtained from and as follows.

16 $$\:\varPsi\:=-{\stackrel{-}{V}}_{12}{\stackrel{-}{V}}_{22}^{-1}$$

#### Step 6

Calculate the eigenvalues of the rotation matrix Ψ. The frequency *f*_*i*_ and damping factor α_*i*_ of all modes of the oscillation signal can be calculated as follows.

17 $$\:{f}_{i}=\frac{{\text{tan}}^{-1}\left(\frac{Im\left({\lambda\:}_{\varPsi\:i}\right)}{Re\left({\lambda\:}_{\varPsi\:i}\right)}\right)}{2\pi\:{T}_{S}},$$

18 $$\:{{\upalpha\:}}_{\text{i}}=-\frac{\text{ln}\left|{\lambda\:}_{\varPsi\:i}\right|}{{T}_{s}},$$

where *λ*_*Ψi*_ are the eigenvalues of the rotation matrix* Ψ*, *T*_*s*_ is the signal sampling time, and ∀_i_= 1, 2, 3, …. 2P.

#### Step 7

Using the eigenvalues of the rotation matrix *λ*_*Ψi*_, Vandermonde matrix *λ*_*v*_ is constructed as follows.

19 $$\:{\lambda\:}_{v}=\left[\begin{array}{cccc}1&\:1&\:\cdots\:&\:1\\\:{\lambda\:}_{1}&\:{\lambda\:}_{2}&\:\cdots\:&\:{\lambda\:}_{p}\\\:\vdots\:&\:\vdots\:&\:\ddots\:&\:\vdots\:\\\:{\lambda\:}_{1}^{J-1}\:&\:{\lambda\:}_{2}^{J-1}&\:\cdots\:&\:{\lambda\:}_{p}^{J-1}\end{array}\right]\:,$$

The original amplitude and initial phase angle can be obtained by the least squares method, where $\:\widehat{X}={\lambda\:}_{v}.{C}_{i}.$

#### Step 8

Calculate where *C*_*i*_ and are column vectors that can be represented as follows.

$$\:{C}_{i}={\left[\begin{array}{cccc}{C}_{1}&\:{C}_{2}&\:\cdots\:&\:{C}_{p}\end{array}\right]}^{T},$$

20 $$\:\widehat{X}={\left[\begin{array}{cccc}\widehat{x}\left(0\right)&\:\widehat{x}\left(1\right)&\:\cdots\:&\:\widehat{x}\left(J-1\right)\end{array}\right]}^{T}.$$

#### Step 9

The original amplitude and initial phase angle of each *i*^*th*^ component can be obtained as follows.

21 $$\:{A}_{i}=2\left|\begin{array}{c}{C}_{i}\end{array}\right|,\:{\theta\:}_{i}={\text{tan}}^{-1}\left(\frac{Im\left({C}_{i}\right)}{Re\left({C}_{i}\right)}\right).$$

### The proposed real-time stability assessment approach

**Fig. 2:**
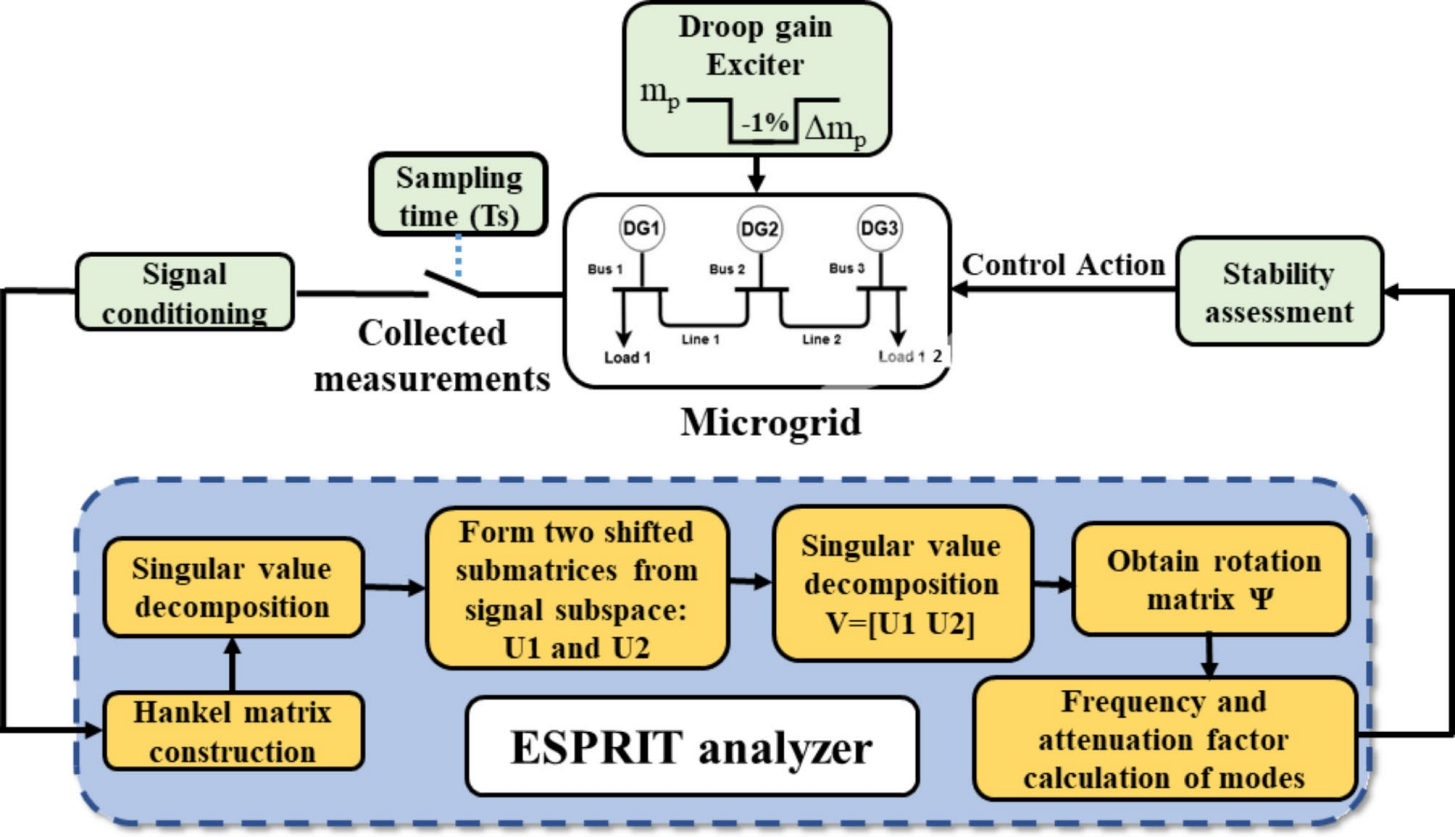
Local stability assessment ESPRIT-based algorithm block diagram.

As outlined in section “Small-signal model-based analysis”, the state space matrix *A*_*mg*_ represents the small-signal model at a specific operating point. On the contrary, the proposed approach employs ESPRIT technique to obtain the microgrid’s most dominant modes in real-time. The block diagram of the suggested ESPRIT estimator is illustrated in Fig. 2, where the droop gain exciter disturbs the active power droop gain for the selected DG for a very short duration (300 msec in this study). The system will respond to this small intentional perturbation and will start oscillating. After the perturbation removal, the real-time measurements are collected and pre-processed, where raw sensors data is filtered to remove noise and finally fed into the ESPRIT analyzer which follows the steps mentioned in section “ESPRIT analysis background” to get the dominant modes of the system. In industrial systems, the proposed algorithm can be integrated into the local controller of each DG unit by using commercially available digital signal processors (DSPs) or microcontrollers. These platforms are well-suited for real-time implementation due to their high computational efficiency and ability to handle the relatively low computational burden of ESPRIT algorithm. Therefore, the algorithm can operate as an add-on module to the local droop controller to enable the real-time monitoring of the system’s stability margin.

As per the analysis in^31^, the low-frequency modes are mainly affected by the DG angle, active power, and reactive power. Hence, for a strictly local estimation procedure, the proposed algorithm uses only one locally measured DG variable. Assessing the microgrid stability is carried out by evaluating the location in the s-plane of the dominant modes (represented by the exponent *(*$\:{\alpha\:}_{i}\pm\:\:j2\pi\:{f}_{i}$*)*) that are predicted by the ESPRIT analysis of the selected measurement signal. The real-time stability assessment is repeated based on an expectation of how frequently unexpected contingency events occur such as the temporary DGs disconnection or sudden variations of loads in the microgrid.

### Simulation results

**Fig. 3:**
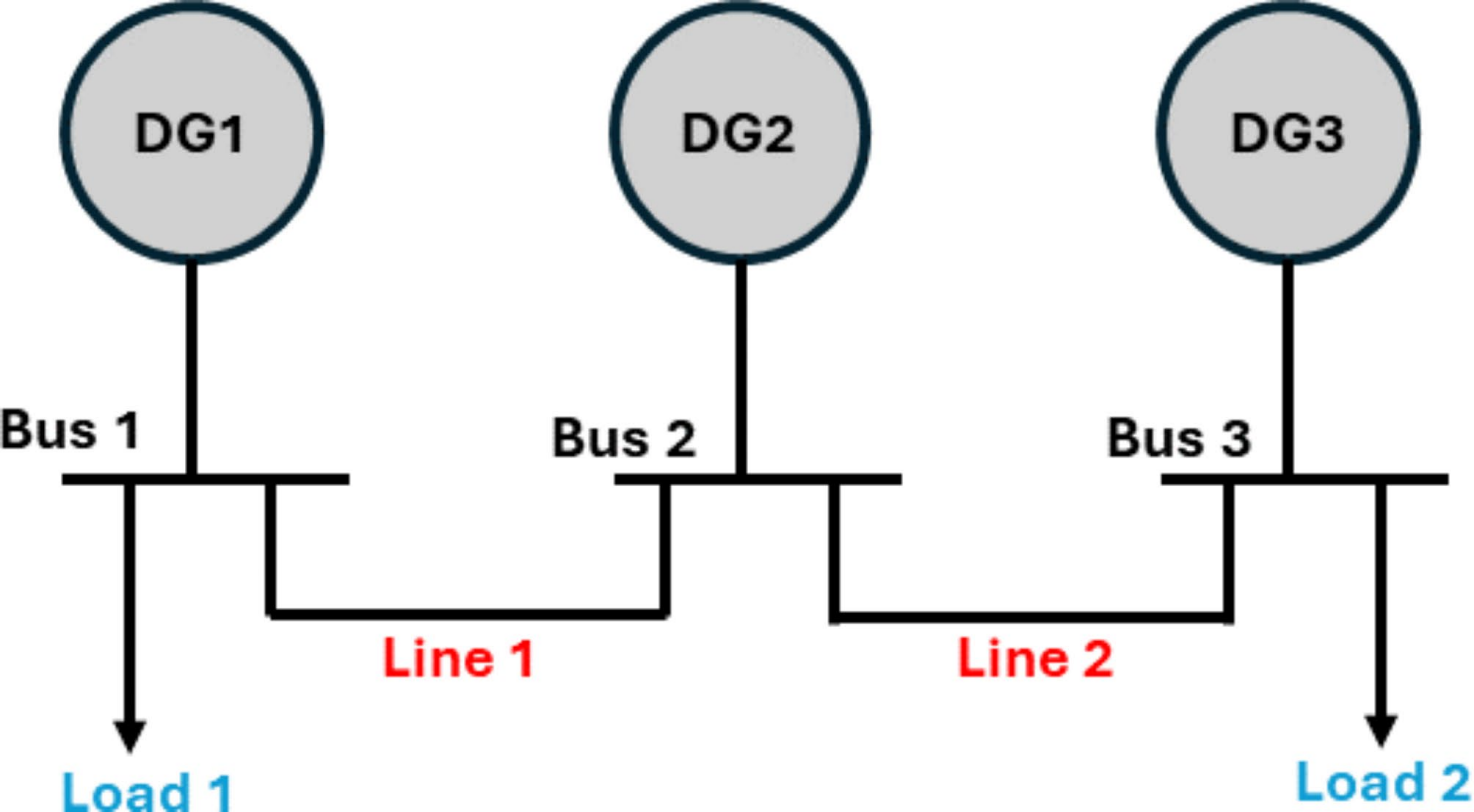
Single-line diagram for the 3-bus benchmark.

**Fig. 4:**
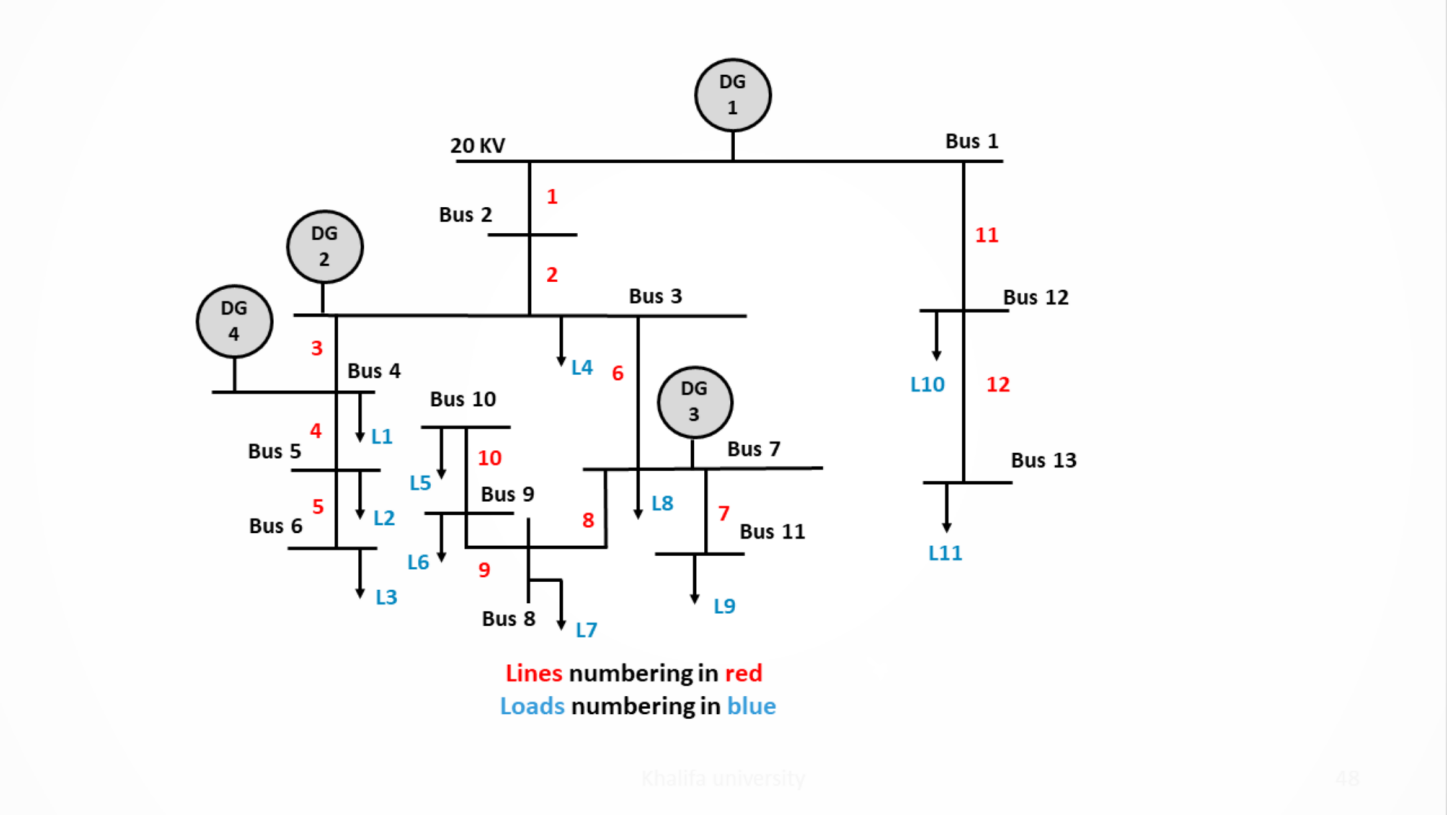
Single-line diagram for the modified CIGRE MV benchmark.

Two benchmark systems are introduced in this paper to validate the proposed ESPRIT algorithm. The 3-bus network which was developed in^31^ and a modified version of the CIGRE MV benchmark which is derived from the German MV distribution network introduced in^38^. The 3-bus microgrid is depicted in Fig. 3 and is comprised of three identical DGs of the same rating (10 KVA) connected via two connecting lines and supplying two loads. The microgrid has a nominal frequency of 50 Hz and a nominal phase voltage of 220 V. The system base power is selected to be 4 KVA. The detailed parameters of the given network are listed in Table 1. On the other hand, the modified version of the CIGRE system has a rated voltage of 20 KV and a nominal frequency of 60 Hz. As shown in Fig. 4, the CIGRE system includes two subnetworks where lines 11 and 12 are underground cables and the rest of the lines in the network are overhead lines. The microgrid has four identical DG units of 2 MVA each, 12 connecting lines, and 11 loads. Network line parameters are provided in the appendix of^39^. The total network load is 4.9 MW and 1.7 MVAR and the system base power is selected as 2 MVA.

**Fig. 5:**
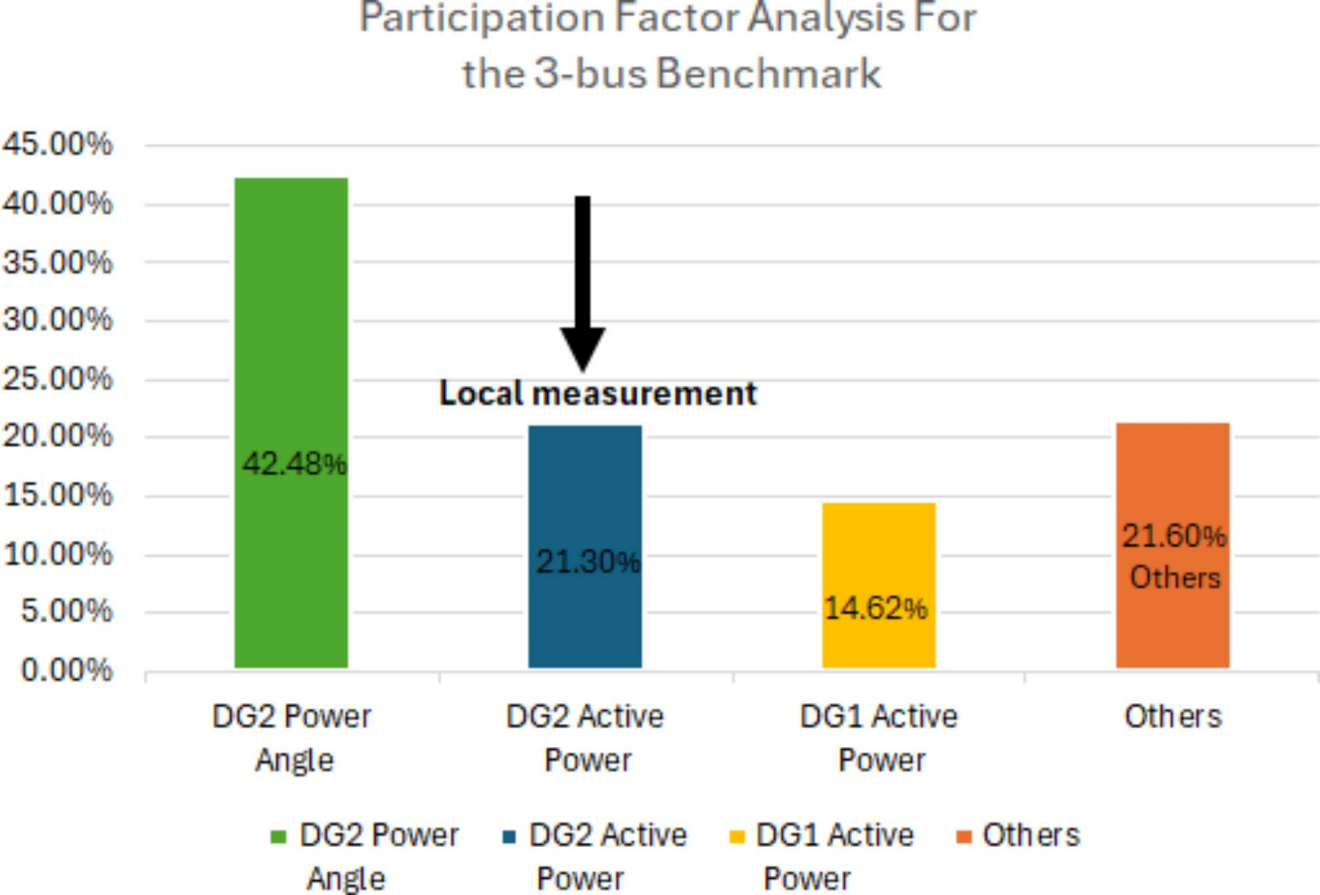
Illustrative bar chart for participation factor analysis for the 3-bus benchmark.

**Fig. 6:**
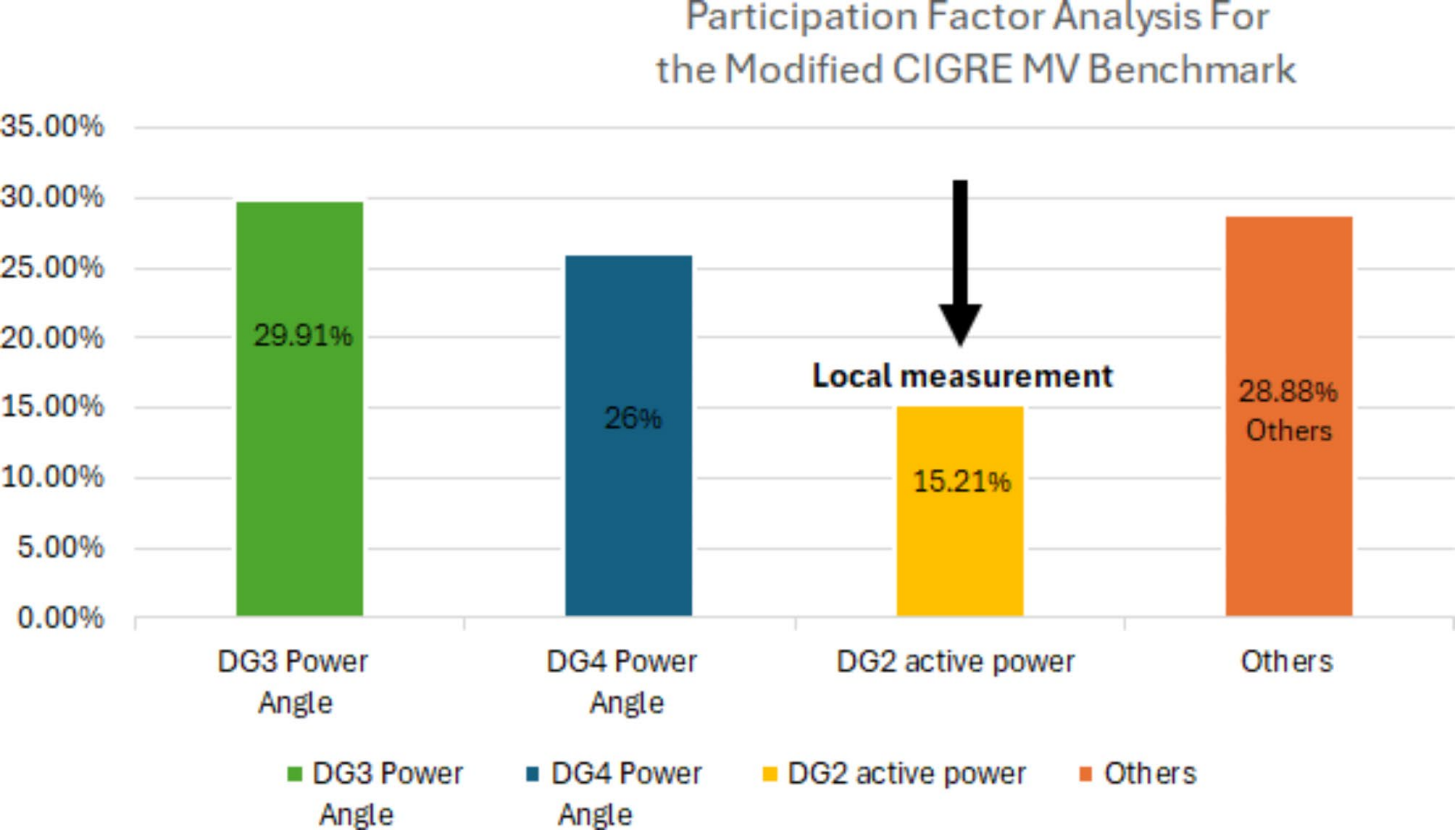
Illustrative bar chart for participation factor analysis for the modified CIGRE MV benchmark.

To select the measurements that have the highest participation in the dominant modes, participation factor analysis was performed. Table 2 illustrates the participation percentages of the state variables for the 3-bus microgrid when m_p_=1.1 × 10^− 4^ and for the modified CIGRE microgrid when m_p_=0.7 × 10^− 7^. It shows that DG2 power angle and active power have the highest percentage of participation for the first system and for the CIGRE system, DG3 and DG4 power angles have the largest participation ratio followed by DG2 active power. These results are explicitly presented in Figs. 5 and 6. Although changes in the active power droop gain might affect the participation factor percentages, these changes are minor and can be neglected. To attain local estimations and avoid the use of PMUs and their associated communication channels, power angles will not be considered in this work and the estimation signal will be taken locally from the perturbed DG. The measurement data were down-sampled to minimize the processing time and to ensure that the estimation process was completed within milliseconds. Across all case studies, the maximum estimation time recorded was 0.0778 s, utilizing an 11th Gen Intel^®^ Core™ i7-1165G7 processor operating at 2.80 GHz. This computational time is determined by the key operations involved in the proposed algorithm, which include the construction of the Hankel matrix from the measurement data, the Singular Value Decomposition (SVD) of the Hankel matrix, and the computation of eigenvalues to estimate the system’s dominant modes. This suggests that the proposed approach is feasible for deployment in practical microgrid systems and real-time applications.

The robustness of the proposed ESPRIT algorithm is validated and tested through four different case studies in the next subsections. In the first case study, the impact of variations of the active power droop gains on ESPRIT estimation accuracy is investigated, while in the second case study, the impact of variation of lines’ X/R ratio is evaluated. In the third case study, the impact of signal noise on the accuracy of the proposed estimator is studied. Finally, the performance of the algorithm is evaluated under large load changes and topological disturbances. In all cases, the estimation was performed locally using the real-time measurements of DG2 active power which are assumed to be instantaneously available for mode estimation. The accuracy of the estimation for the proposed approach was checked and validated by comparing the estimated modes to the eigenvalues derived from the small-signal analysis.

**Table 1:** Microgrid parameters for the 3-bus benchmark.

Parameter	Value	Parameter	Value
$\:{\text{R}}_{\text{f}}$ (Ω)	1e^− 1^	$\:{\text{R}}_{\text{l}\text{i}\text{n}\text{e}2}$(Ω)	35e^− 2^
$\:{\text{L}}_{\text{f}}$ (H)	1.35e^− 3^	$\:{\text{L}}_{\text{i}\text{n}\text{e}2}$ (H)	1.85e^− 3^
$\:{\text{C}}_{\text{f}}$ (F)	50e^− 6^	$\:{\text{R}}_{\text{l}\text{o}\text{a}\text{d}1}$(Ω)	25
$\:{\text{R}}_{\text{C}}\:$(Ω)	0.03	$\:{\text{L}}_{\text{l}\text{o}\text{a}\text{d}1}$ (H)	1e^− 2^
$\:{\text{L}}_{\text{C}}$ (H)	0.35e^− 3^	$\:{\text{R}}_{\text{l}\text{o}\text{a}\text{d}2}$(Ω)	25
$\:{\text{R}}_{\text{l}\text{i}\text{n}\text{e}1}$(Ω)	23e^− 2^	$\:{\text{L}}_{\text{l}\text{o}\text{a}\text{d}2}$ (H)	1e^− 2^
$\:{\text{L}}_{\text{l}\text{i}\text{n}\text{e}1}$ (H)	0.35e^− 3^	Voltage (V)	381
$\:\text{X}/{\text{R}}_{\text{l}\text{i}\text{n}\text{e}1}$	0.477	$\:\text{X}/{\text{R}}_{\text{l}\text{i}\text{n}\text{e}2}$	1.66

**Table 2:** Participation factor analysis for the 3-bus benchmark at m_p_=1.1 × 10^− 4^ and the modified CIGRE MV benchmark at m_p_=0.7 × 10^− 7^.

System	Measurement	Participation factor %	Type of measurement
3-bus microgrid	DG2 power angle	42.48	Communication-based
	**DG2 active power**	**21.3**	**Local**
	DG1 active power	14.62	Local
	Others	21.6	–
Modified CIGRE MV microgrid	DG3 power angle	29.914	Communication-based
	DG4 power angle	26	Communication-based
	**DG2 active power**	**15.208**	**Local**

Significant values are in bold.

### Impact of variation of the active power droop gain on the estimation accuracy of the proposed approach

**Fig. 7:**
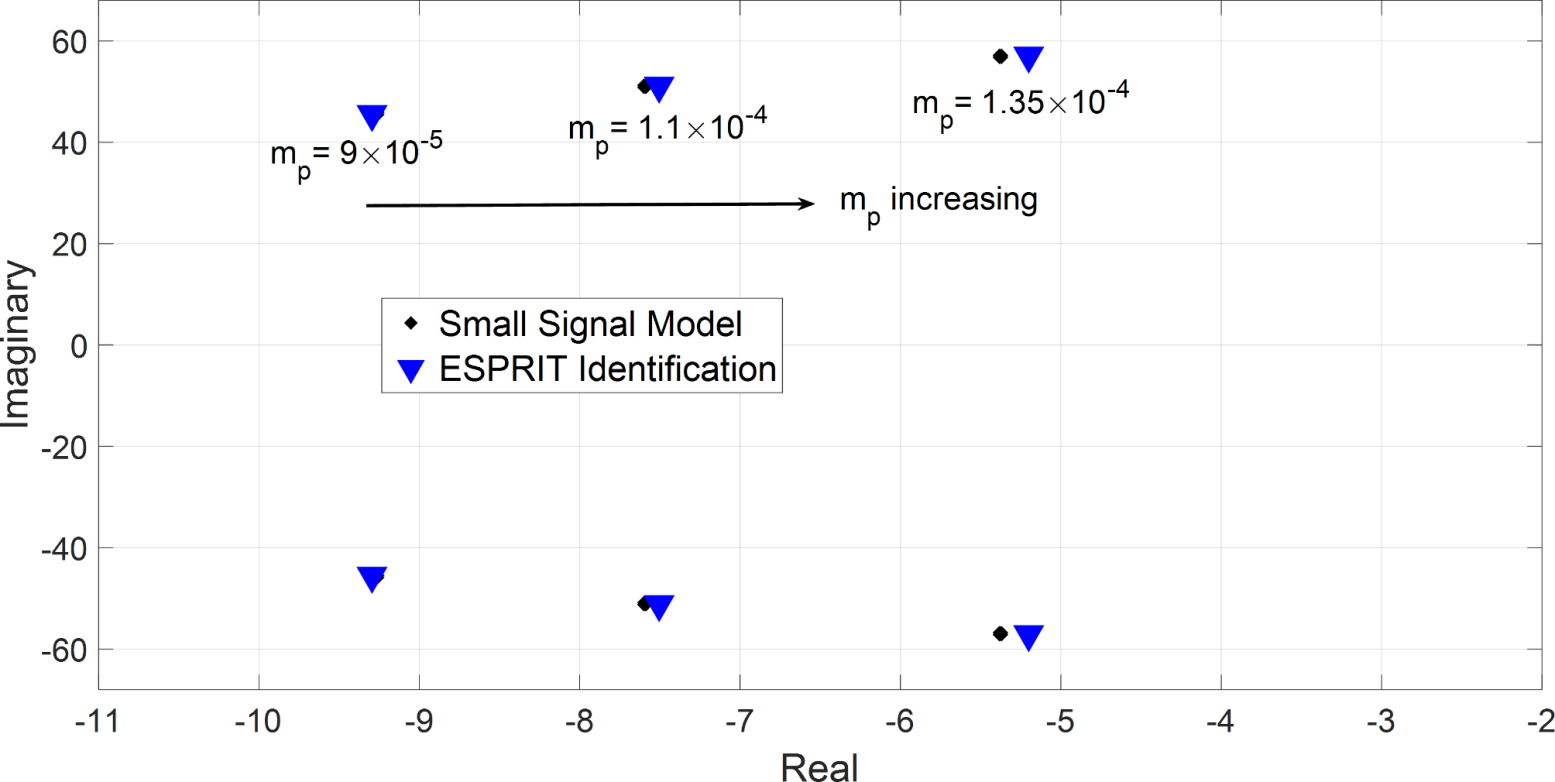
Analytically derived modes versus estimated modes for the 3-bus benchmark at different active power droop gains, m_p_= (0.9, 1.1, 1.35) ×10^− 4^.

The proposed ESPRIT estimator was applied to the 3-bus microgrid and the modified CIGRE MV system, both tested under three different active power droop gains. Additionally, to further substantiate its robustness and reliability, ESPRIT estimator was evaluated on a 34-bus microgrid^40^, representing a larger and more complex microgrid, to rigorously assess its estimation accuracy. In all cases, the active power droop gain of DG2 was perturbed with a negative perturbation of Δm_p_= -1% for a duration of 300 msec, then the original $\:{\text{m}}_{\text{p}}$ was restored. The percentage of perturbation Δm_p_ and its duration were minimized to the least possible level that maintains an accurate estimation of the system’s most dominant modes. At each droop value, the estimation was performed using DG2 active power to evaluate the estimation accuracy of the proposed ESPRIT algorithm. In Fig. 7, the estimated most dominant modes for the 3-bus microgrid are plotted versus the analytically derived modes from the small-signal analysis at m_p_=9 × 10^− 5^, m_p_=1.1 × 10^− 4^, and m_p_=1.35 × 10^− 4^. It is evident that as increases, the dominant modes are shifted towards the right-hand side, causing a reduction in the network stability margin. Additionally, the estimated and analytically derived modes are very close to each other for different values of active power droop gains which demonstrates the high accuracy of estimation for the proposed algorithm. Table 3 lists the estimated dominant modes for the three above-stated droop gains. The estimation accuracy of ESPRIT technique is also compared to Prony, Matrix Pencil, and SID techniques. In all cases, the estimation of ESPRIT method has a percentage error of less than 3.25%in the real part and 0.254% in the imaginary part which is a high accuracy given that it is a local estimator that does not require any communication channels. Prony algorithm identifies the most dominant modes with a percentage error of up to 9.87% in the real part and 4% in the imaginary part. The results of Matrix Pencil are very close to those obtained by Prony algorithm. Finally, SID technique was applied to estimate the dominant modes for the above-mentioned droop gains with a maximum error of 8% in the real part and 3.41% in the imaginary part.

**Fig. 8:**
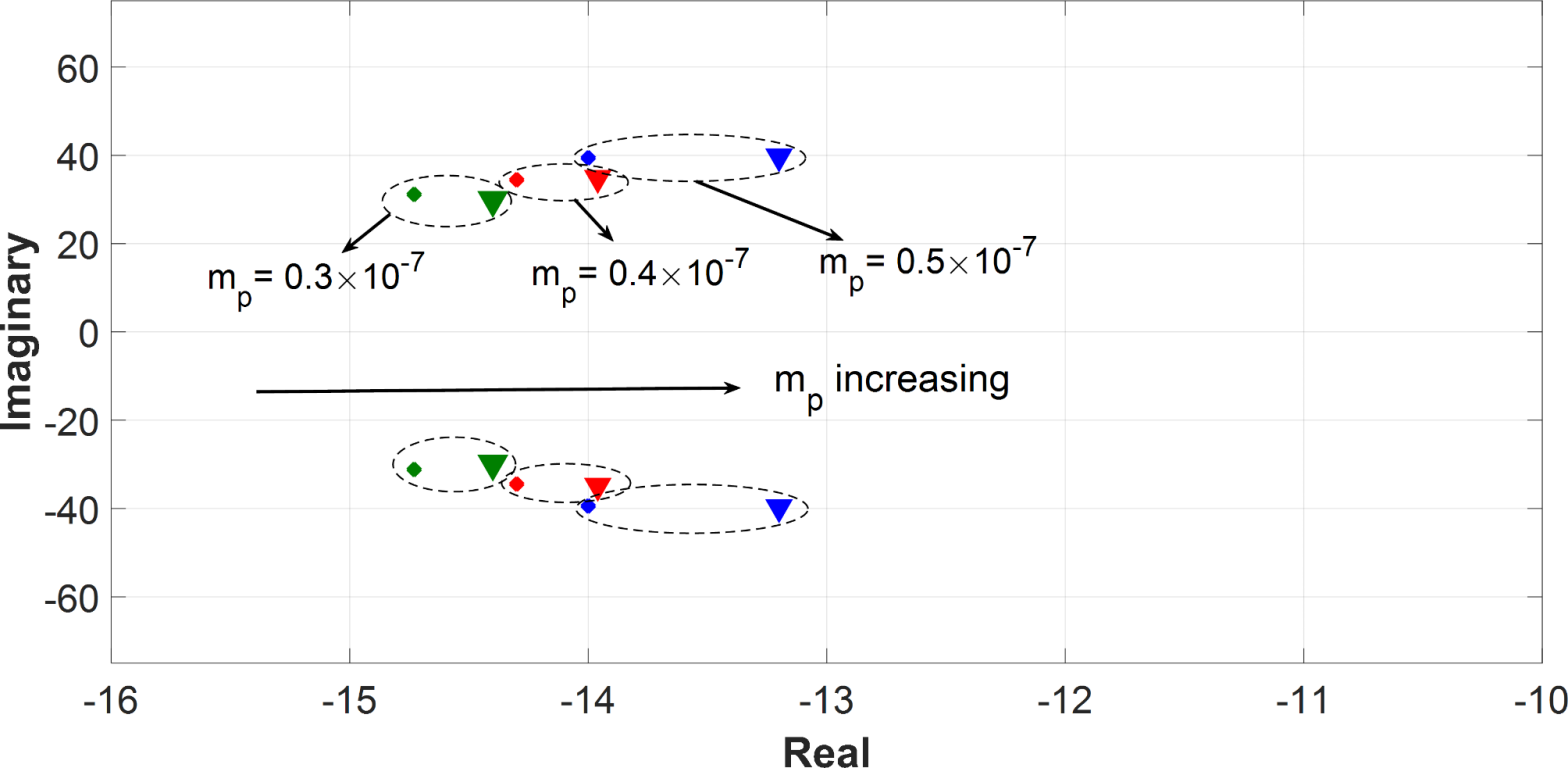
Analytically derived modes versus estimated modes for the modified CIGRE benchmark at different active power droop gains, m_p_= (0.3, 0.4, 0.5) ×10^− 7^.

**Fig. 9:**
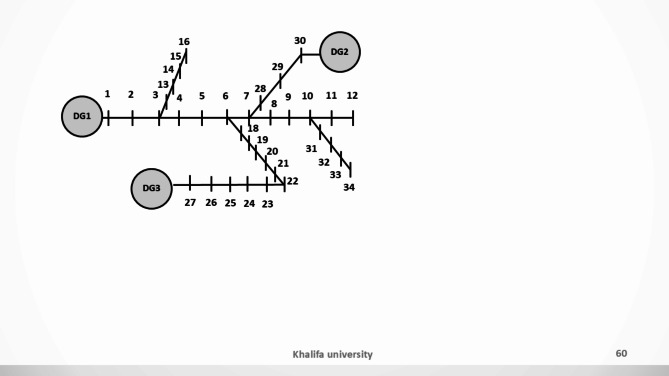
Single-line diagram for the 34-bus benchmark.

For further verification of the robustness of the proposed ESPRIT estimator, a larger system was adopted, and the algorithm was tested on the modified CIGRE microgrid at three values of m_p_ (m_p_=0.3 × 10^− 7^, m_p_=0.4 × 10^− 7^, and m_p_=0.5 × 10^− 7^). As listed in Table 4, ESPRIT algorithm estimated the most dominant modes with a percentage error of less than 5.71% in the real part and 4.17% in the imaginary part. Compared to SID, Prony, and Matrix Pencil techniques which have a percentage error of up to 10.25% in the real part and 14.41% in the imaginary part, ESPRIT technique demonstrates its superiority over the other methods in terms of the estimation accuracy for different values of active power droop gains. Figure 8 plots the estimated most dominant modes versus the analytically derived modes at m_p_=0.3 × 10^− 7^, m_p_=0.4 × 10^− 7^, and m_p_=0.5 × 10^− 7^ for the modified CIGRE microgrid. At the final stage of the evaluation, the performance of the ESPRIT estimator was tested on a larger and more complex microgrid configuration. The 34-bus radial distribution network presented in^40^ was modified to form a droop-controlled inverter-based microgrid, incorporating three identical distributed generation (DG) units, each rated at 2 MVA and connected to buses 1, 27, and 30 as depicted in Fig. 9. The network operates at a rated voltage of 11 kV, with a total installed load of 5.4 MVA and an average power factor of 0.85. Table 5 summarizes the results of the ESPRIT evaluations conducted on the 34-bus microgrid, with dominant modes estimated at m_p_=0.5 × 10^− 7^, m_p_ = 0.8 × 10^− 7^, and m_p_​=1.1 × 10^− 7^. Across all specified droop gain values, the ESPRIT method achieved percentage errors of less than 2.38% in the real part and 2.162% in the imaginary part. In comparison, alternative techniques such as Prony, Matrix Pencil, and SID exhibited higher errors, with maximum errors reaching 5.52% in the real part and 5.1733% in the imaginary part. These results underscore the robustness and superior accuracy of the ESPRIT method, even when applied to large and complex microgrid systems. Since it is inherently a data-driven technique, the proposed ESPRIT algorithm can operate independently of the system size or topology which ensures the algorithm’s scalability across systems with multiple distributed generators or more complex configurations.

**Fig. 10:**
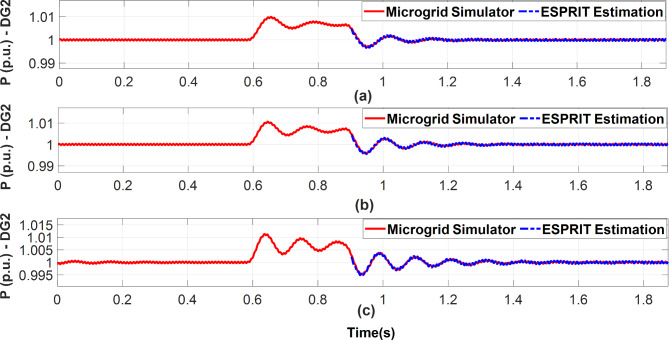
Estimated DG2 active power versus real measurements for the 3-bus microgrid simulator while excitation of DG2 active power droop gain. **(a)** m_p_=9 × 10^− 5^. **(b)** m_p_=1.1 × 10^− 4^**(c)** m_p_=1.35 × 10^− 4^.

**Fig. 11:**
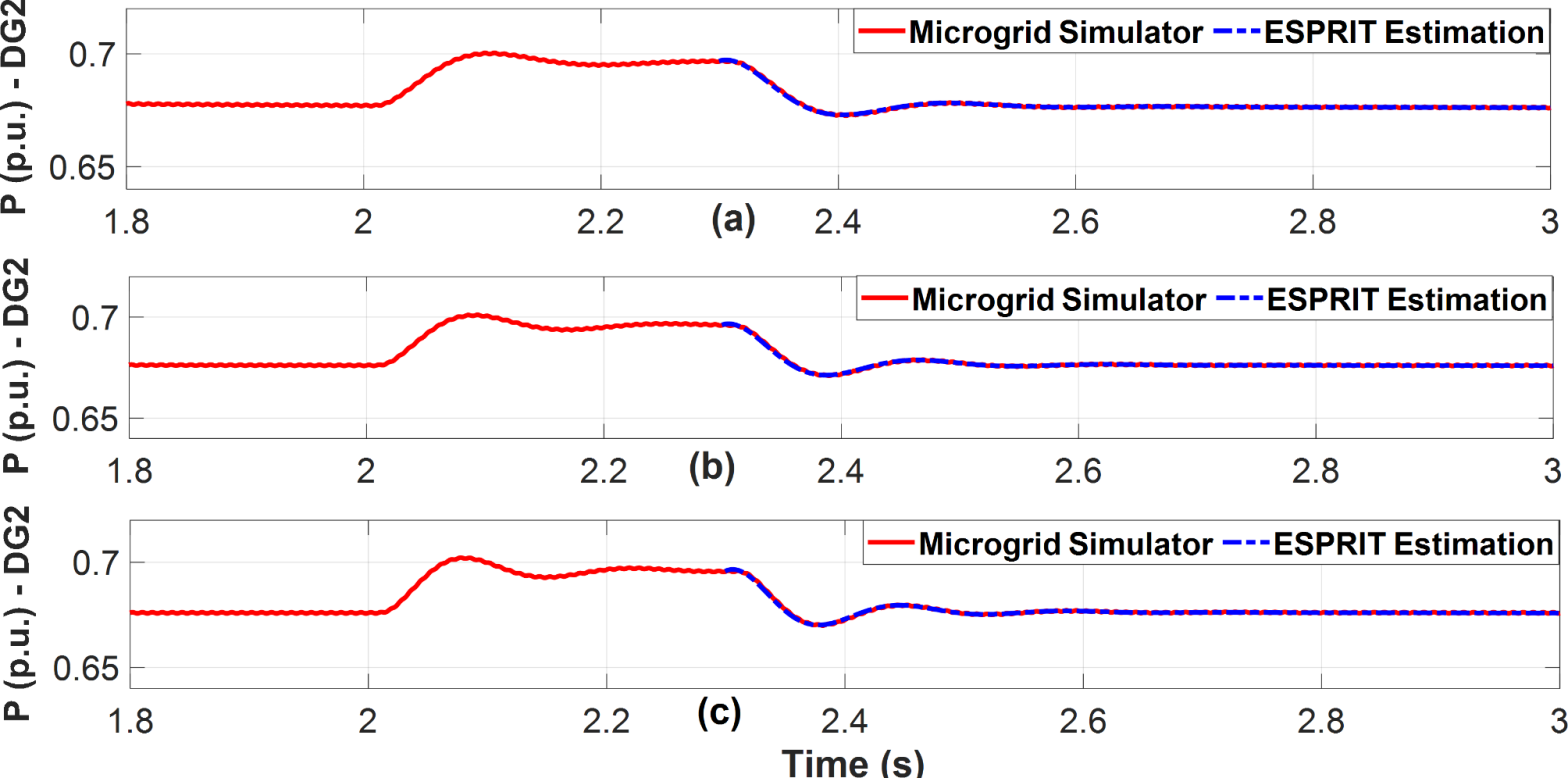
Estimated DG2 active power versus real measurements for the modified CIGRE microgrid while excitation of DG2 active power droop gain. (**a**) m_p_=0.3 × 10^− 7^ (**b**) m_p_=0.4 × 10^− 7^ (**c**) m_p_=0.5 × 10^− 7^.

For all presented benchmarks, the Kurtosis measure was calculated for each measurement data vector at each droop gain, and it shows a large value (K > 3) which implies that the distribution of data holds extreme values (observations) thus, enabling ESPRIT to do accurate estimations for the system’s dominant modes. Additionally, the real-time measurements of DG2 active power are plotted against the estimated signals as depicted in Figs. 10 and 11, and they explicitly reveal excellent fitting between them. In both figures, during the short perturbation period and due to the negative perturbation on DG2 active power droop gain, DG2 provides more power than the other DGs in the microgrid. Afterward, the steady state power is restored after the removal of the perturbation.

**Table 3:** Percentage error of estimated dominant modes in the 3-bus benchmark for different active power droop gains using ESPRIT, Prony, Matrix Pencil, and SID techniques.

m_p_×10^− 4^	System modes	ESPRIT			Kurtosis measure	m_p_×10^− 4^	Prony		
		Estimated modes	% Error				Estimated modes	% Error	
			Real	Imag				Real	Imag
0.9	−9.26 ± 45.674i	−9.22 ± 45.79i	0.431	0.254	12.951	0.9	−10.174 ± 47.504i	9.87	4.0065
						$\:1.1$	−8.2739 ± 52.382i	8.965	2.61
						$\:1.35$	−5.6602 ± 58.355i	5.28	2.4351
1.1	−7.593 ± 51.05i	−7.504 ± 51.16i	1.17	0.215	7.6248	Matrix pencil			
						0.9	−10.174 ± 47.504i	9.87	4.0065
1.35	−5.376 ± 56.97i	−5.201 ± 57.09i	3.25	0.21	5.64	$\:1.1$	−8.2739 ± 52.523i	8.965	2.881
						$\:1.35$	−5.6674 ± 58.356i	5.42	2.43
						Subspace Identification			
						0.9	−9.97 ± 45.72i	7.66	0.1
						$\:1.1$	−8.2 ± 52.794i	7.99	3.41
						$\:1.35$	−5.81 ± 57.22i	8.072	0.438

**Table 4:** Percentage error of estimated dominant modes in the modified CIGRE MV benchmark for different active power droop gains using ESPRIT, Prony, Matrix Pencil, and SID techniques.

m_p_ ×10^− 7^	System modes	ESPRIT			Kurtosis measure	m_p_×10^− 7^	Prony		
		Estimated modes	% Error				Estimated modes	% Error	
			Real	Imag				Real	Imag
0.3	−14.73 ± 31.15i	−14.4 ± 29.85i	2.24	4.17	9.357	0.3	−13.224 ± 33.064i	10.22	6.14
						0.4	−13.109 ± 39.416i	8.328	14.41
						0.5	−12.603 ± 44.854i	9.97	13.78
0.4	−14.3 ± 34.45i	−13.96 ± 34.85i	2.37	1.16	8.9	Matrix pencil			
						0.3	−13.22 ± 33.068i	10.25	6.157
0.5	−14 ± 39.42i	−13.2 ± 39.72i	5.71	0.76	6.92	0.4	−13.11 ± 39.416i	8.321	14.41
						0.5	−12.618 ± 44.854i	9.87	13.78
						Subspace identification			
						0.3	−13.987 ± 32.2811i	5.04	3.63
						0.4	−13.321 ± 37.362i	6.84	8.45
						0.5	−12.818 + 40.73i	8.44	3.32

**Table 5:** Percentage error of estimated dominant modes in the 34-bus microgrid for different active power droop gains using ESPRIT, Prony, Matrix Pencil, and SID techniques.

m_p_ ×10^− 7^	System modes	ESPRIT			Kurtosis measure	m_p_×10^− 7^	Prony		
		Estimated modes	% Error				Estimated modes	% Error	
			Real	Imag				Real	Imag
0.5	−5.63 ± 10.612i	−5.5748 ± 10.67i	0.98	0.546	4.0043	0.5	−5.4713 ± 10.23i	2.818	3.59
						0.8	−4.3018 ± 13.559i	0.98	2.17
						1.1	−3.3729 ± 15.928i	5.52	1.019
0.8	−4.26 ± 13.27i	−4.282 ± 13.557i	0.51	2.162	5.3532	Matrix pencil			
						0.5	−5.4674 ± 10.227i	2.889	3.627
1.1	−3.57 ± 16.092i	−3.485 ± 16.24i	2.38	0.919	5.5959	0.8	−4.3021 ± 13.559i	0.988	2.17
						1.1	−3.3728 ± 15.928i	5.52	1.019
						Subspace identification			
						0.5	−5.716 ± 11.161i	1.5275	5.1733
						0.8	−4.336 ± 13.706i	1.784	3.285
						1.1	−3.738 ± 16.462i	4.705	2.299

### Impact of variation of lines’ X/R ratio on the estimation accuracy of the proposed approach

In this section, the suggested ESPRIT-based stability assessment approach is tested to demonstrate its ability to handle changes in network parameters, such as the changes in the lines’ X/R ratio in the microgrid. Generally, the system’s critical oscillatory modes rely on the droop gains, the number of inverters, and the lines’ X/R ratio^41,42^. Additionally, microgrids are usually low-voltage networks with a limited geographical area and short lines. Based on this, they are considered distribution networks that are characterized by low values of the X/R ratio. The X/R ratio varies depending on the MG size and type of the network. In this subsection, the lines’ X/R ratio was varied, and the estimation accuracy of the proposed ESPRIT algorithm was tested under these variations for different values of 55. Starting with the 3-bus system, four case studies were studied in this section as follows: (a) Line1 X/R ratio increased by 20%, (b) Line2 X/R ratio increased by 20%. (c) Line1 X/R ratio decreased by 20%, and (d) Line2 X/R ratio decreased by 20%.

Table 6 presents the percentage errors of identifying the most dominant modes for the four case studies compared with the reference case of the system’s original X/R ratio for two values of m_p_ (m_p_=1.1 × 10^− 4^ and m_p_=1.35 × 10^− 4^) in the 3-bus microgrid. ESPRIT method demonstrated a minimal estimation error for various X/R ratios for both lines. As noticed, the maximum percentage error is less than 4.207% in the real part and 0.768% in the imaginary part. It is worth mentioning that the lines with the minimum impedance between the sources have a strong impact on the system stability margin^43^. For more validation of the effectiveness of the proposed algorithm, the test was repeated on the modified CIGRE MV benchmark. As can be seen in Table 7, the most dominant modes were estimated and compared to the analytically derived modes at m_p_=0.7 × 10^− 7^ in four case studies presented as follows: (a) Line6 X/R ratio increased by 50%, (b) Line3 X/R ratio increased by 50%. (c) Line2 X/R ratio decreased by 50%, and (d) Line12 X/R ratio decreased by 50%. In all cases, the percentage error is less than 7.22% in the real part and 6.58% in the imaginary part. Thus, with different variations of the system’s X/R ratio, ESPRIT is still capable of accurately estimating the most dominant modes. Additionally, all cases, in both benchmarks, have a large Kurtosis measure (K > 3) which indicates the presence of non-Gaussian components in the data distribution. In other words, each signal used in the estimation process holds prominent features allowing accurate estimations for the dominant modes. Thus, it can be concluded that the proposed ESPRIT algorithm can accommodate the changes in the microgrid line parameters while maintaining high estimation accuracy for its dominant modes.

**Table 6:** Percentage error in estimating dominant modes at different X/R ratios and active power droop gains for the 3-bus benchmark.

m_*p*_× 10^− 4^	X/*R* ratio	System modes	Estimated modes	% Error Real	% Error Imag	Kurtosis measure
1.1	Unchanged (reference case)	−7.59 ± 51.05i	−7.504 ± 51.16i	1.17	0.215	7.6248
1.35		−5.37 ± 56.97i	−5.201 ± 57.09i	3.25	0.21	5.64
1.1	Line 1 X/R Increased by 20%	−8.56$\:\pm\:$50.05i	−8.508 ± 50.4i	0.607	0.699	9.31
1.35		−6.5544$\:\pm\:$ 56i	−6.686 ± 56.12i	2.0078	0.214	6.6
1.1	Line 2 X/R Increased by 20%	−7.516$\:\pm\:$50.7i	−7.596 ± 50.86i	1.064	0.3155	8.3
1.35		−5.31$\:\pm\:$56.58i	−5.198 ± 56.76i	2.109	0.318	5.7
1.1	Line 1 X/R decreased by 20%	−6.48$\:\pm\:52.04$i	−6.397 ± 52.44i	1.28	0.768	7
1.35		−4.04$\:\pm\:57.87$i	−3.87 ± 58.078i	4.207	0.359	4.56
1.1	Line 2 X/R decreased by 20%	−7.64$\:\pm\:51.54$i	−7.61 ± 51.79i	0.392	0.485	8.33
1.35		−5.38$\:\pm\:57.55$ i	−5.287 ± 57.58i	1.72	0.052	5.73

**Table 7:** Percentage error in estimating dominant modes at different X/R ratios for the modified CIGRE MV benchmark.

m_*p*_× 10^− 7^	X/*R* ratio	System modes	Estimated modes	% Error Real	% Error Imag	Kurtosis measure
0.7	Unchanged (reference case)	−13.33 ± 47.96i	−12.427 ± 48.45i	6.77	1.02	11.67
0.7	Line 6 X/R Increased by 50%	−12.364 ± 52.4i	−12.505 ± 48.95i	1.14	6.583	12.34
0.7	Line 3 X/R Increased by 50%	−13.33 ± 47.96i	−12.394 ± 48.421i	7.02	0.96	11.68
0.7	Line 2 X/R decreased by 50%	−12.08 ± 53.84i	−11.207 ± 53.87i	7.22	0.055	11.343
0.7	Line 12 X/R decreased by 50%	−12.075 ± 53.82i	−11.276 ± 53.735i	6.61	0.157	10.783

### Impact of signal noise on the estimation accuracy of the proposed approach

To further confirm the robustness of the proposed approach, the anti-noise feature is tested under different levels of noise. White Gaussian noise of different levels of SNR was added to the measurements to test the estimation accuracy of the proposed ESPRIT analyzer. White Gaussian noise has a kurtosis of three, like a standard normal distribution [34]. Generally, this noise content can reduce the overall Kurtosis of the signal, causing prominent features of the original signal to be masked. Starting with the 3-bus benchmark, the dominant modes were estimated and compared to the calculated system modes at m_p_=1.1 × 10 − 4 as listed in Table 8. Compared to the noise-free reference case, the percentage error slightly increased at SNR = 40 dB to reach 2.015% in the real part and 1.65% in the imaginary part with a Kurtosis measure equal to 4.82. By increasing the noise content further to SNR = 30, and 25 dB, the estimation error jumps to 7.4% in the real part and 5.85% in the imaginary part. While there is a noticeable reduction in the kurtosis measure, which attained 3.37 at SNR = 25, it remains above 3, the kurtosis of white Gaussian noise. Thus, the original signal still retains prominent features, allowing ESPRIT to accurately estimate the dominant modes. The test was repeated for the modified CIGRE MV benchmark, and the results are presented in Table 9.

The dominant modes were calculated and then estimated at m_p_=0.3 × 10^− 7^ for different levels of SNR. As shown, the percentage error in the real part at SNR = 40 dB is roughly the same as that of the noise-free case, while the imaginary part error slightly increased to 7.83%. At SNR = 30 dB, ESPRIT estimator is still capable of identifying the dominant modes with an error of 6.04% in the real part and 7.02% in the imaginary part while having a Kurtosis measure of 4.699. Finally, at SNR = 25, and with a signal Kurtosis measure that is roughly equal to that of white Gaussian noise (K = 3.0397 for the combined signal), the error remarkably increased to 18.8% in the real part and 16.8% in the imaginary part which implies that the white Gaussian noise is dominating the original signal and explains the impact of noise on concealing signal features which, in turn, complicates the estimation process.

**Table 8:** Percentage error in estimating dominant modes at different levels of SNR for the 3-bus benchmark.

m_*p*_× 10^− 4^	SNR	System modes	Estimated modes	% Error Real	% Error Imag	Kurtosis measure
1.1	Noise-free	−7.593 ± 51.05i	−7.504 ± 51.16i	1.17	0.215	7.6248
	40		−7.44 ± 51.893i	2.015	1.65	4.82
	30		−7.97 ± 50.132i	4.96	1.79	3.886
	25		−7.031 ± 48.063i	7.4	5.85	3.37

**Table 9:** Percentage error in estimating dominant modes at different levels of SNR for the modified CIGRE MV benchmark.

m_*p*_× 10^− 7^	SNR	System modes	Estimated modes	% Error Real	% Error Imag	Kurtosis measure
0.3	Noise-free	−14.73 ± 31.15i	−14.4 ± 29.85i	2.24	4.17	9.357
	40		−14.401 ± 28.71i	2.23	7.833	7.26
	30		−13.84 ± 28.962i	6.04	7.02	4.699
	25		−11.96 ± 25.914i	18.8	16.8	3.0397

### ESPRIT performance evaluation under large load changes and topological disturbances

This section presents an evaluation of the proposed algorithm’s performance under various dynamic disturbances, including large load changes and topological reconfiguration, with a focus on their impact on the microgrid’s stability margin. In the 3-bus benchmark system, a static RL load of 12 kW and 17 kVAR was suddenly connected to bus 1, and the dominant modes were estimated before and after the disturbance using ESPRIT algorithm at m_p_=0.63 × 10^− 4^. The results, shown in Table 10, indicate that the maximum estimation error is 4.82% in the real part and 1.826% in the imaginary part which demonstrates the high accuracy of estimation for both pre- and post-disturbance conditions. Additionally, despite the considerable load increase, the stability margin was minimally impacted.

To thoroughly investigate the robust performance of ESPRIT algorithm, a dynamic network reconfiguration scenario was implemented on the modified CIGRE benchmark system shown in Fig. 4. This scenario involves disconnecting DG4, disconnection of line 1 between bus 1 and bus 2, and connecting the normally-open tie line between bus 3 and bus 12. The dominant modes were estimated before and after the reconfiguration at m_p_=1.25 × 10^− 7^. As summarized in Table 11, before the reconfiguration, the modes were located at − 4.984 ± 73.39i, with estimation errors of 4.06% in the real part and 1.71% in the imaginary part. Following the reconfiguration, the system displayed an enhanced stability margin, with the modes shifting to − 9.1332 ± 48.818i and a reduction in estimation errors to 3.94% in the real part and 1.037% in the imaginary part. These results further validate the ESPRIT algorithm’s robustness, confirming its effectiveness in accurately estimating dominant modes under significant load changes and topological reconfigurations, thus proving its reliability for practical microgrid stability analysis.

**Table 10:** Percentage error in estimating dominant modes for a large load change in the 3-bus benchmark.

m_*p*_×10^− 4^	Disturbance	System modes	Estimated modes	% Error Real	% Error Imag	Kurtosis measure
0.63	Before step load increase at bus 1	−10.91 ± 36.13i	−10.39 ± 35.47i	4.766	1.826	11.1551
	After step load increase at bus 1	−11.2 ± 35.24i	−10.66 ± 34.625i	4.82	1.745	11.2264

**Table 11:** Percentage error in estimating dominant modes for a reconfiguration scenario in the modified CIGRE MV benchmark.

m_*p*_×10^− 7^	Disturbance	System modes	Estimated modes	% Error Real	% Error Imag	Kurtosis measure
1.25	Before network reconfiguration	−5.1954 ± 74.667i	−4.984 ± 73.39i	4.06	1.71	9.4816
	After network reconfiguration	−9.508 ± 48.317i	−9.1332 ± 48.818i	3.94	1.037	6.831

### Real-time validation of ESPRIT performance using OPAL-RT

**Fig. 12:**
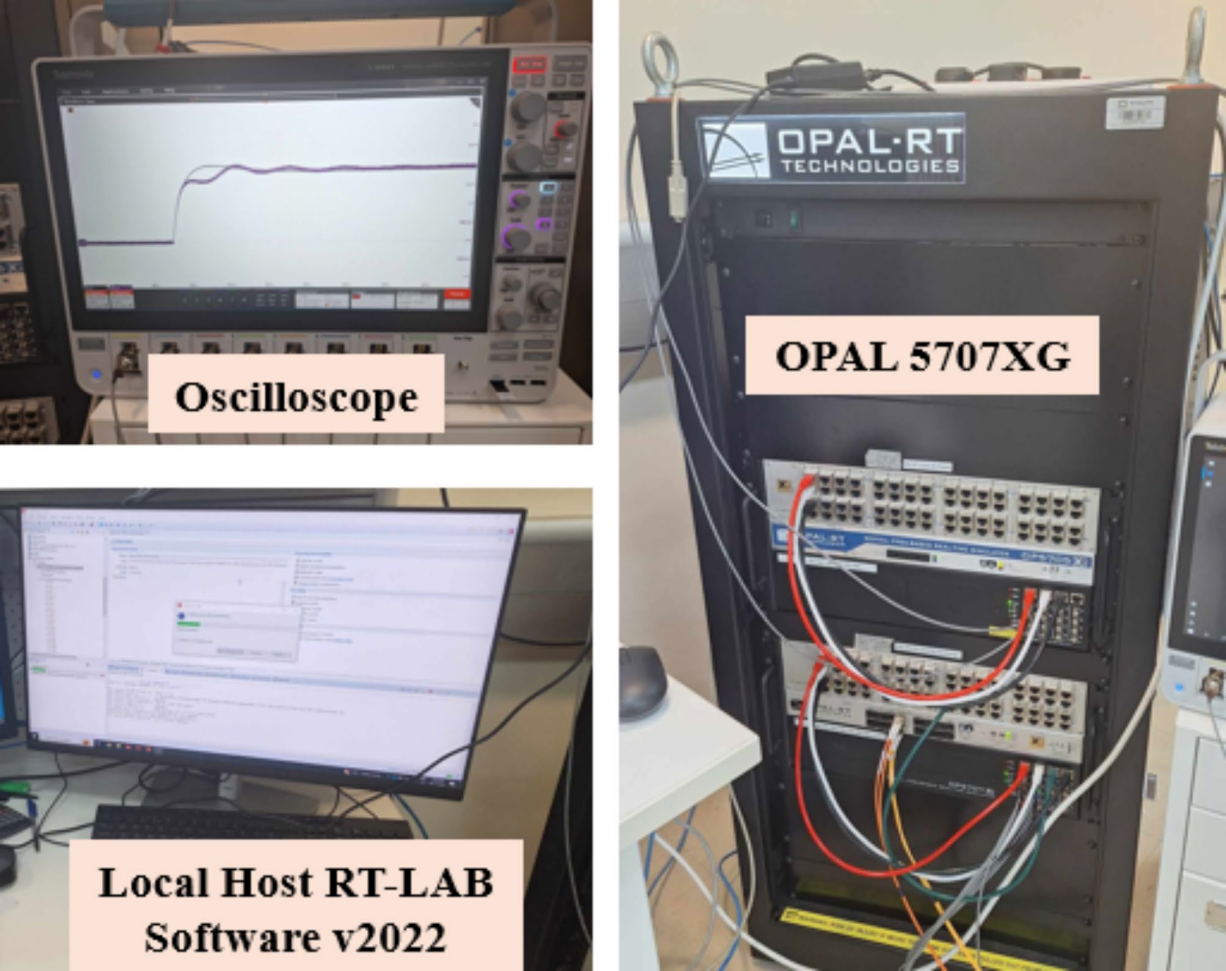
CIL experimental setup.

**Fig. 13:**
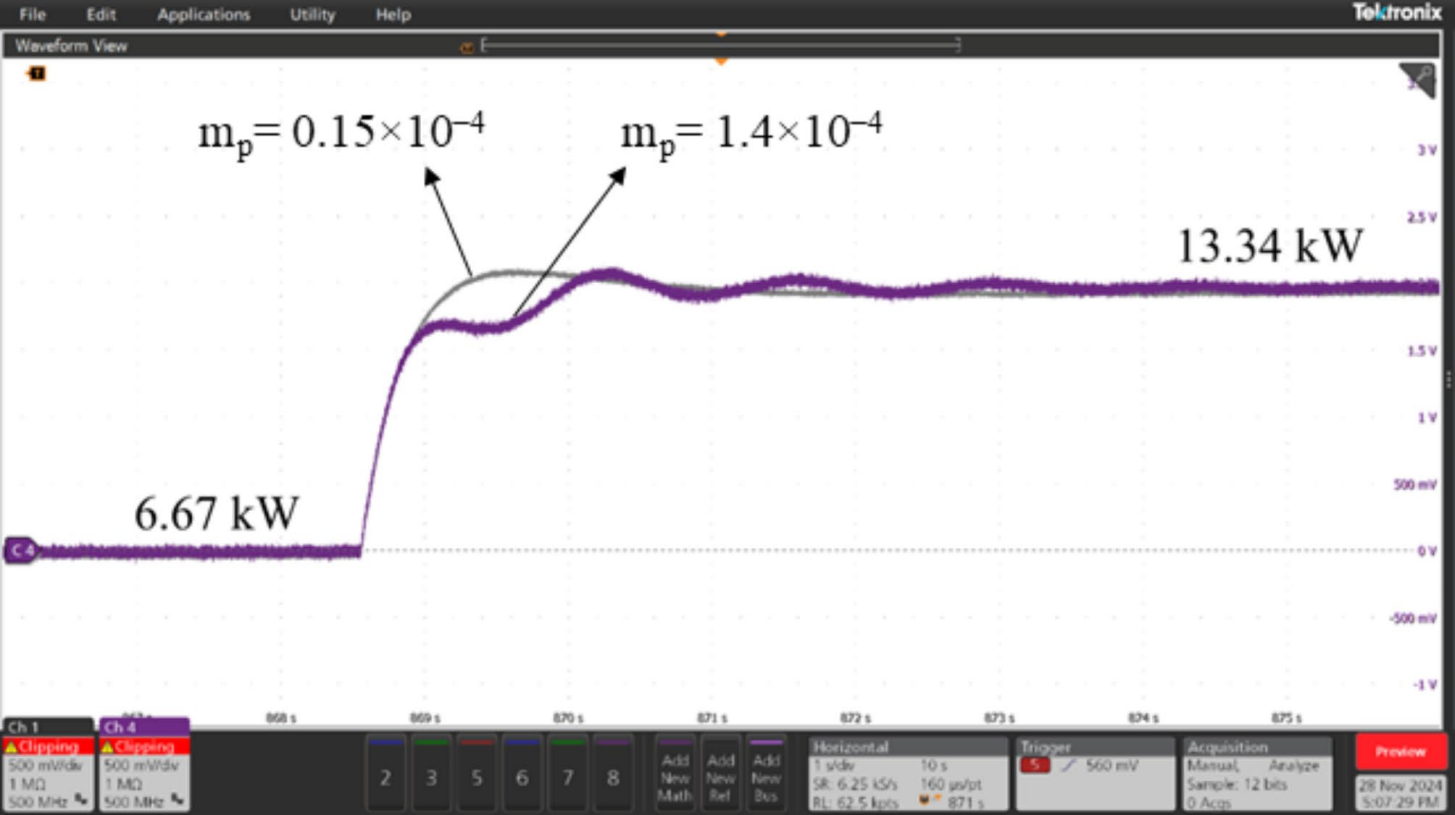
Transient response for DG1 active power at m_p_=0.15 × 10^− 4^ and mp = 1.4 × 10^− 4^ after a step load increase.

**Fig. 14:**
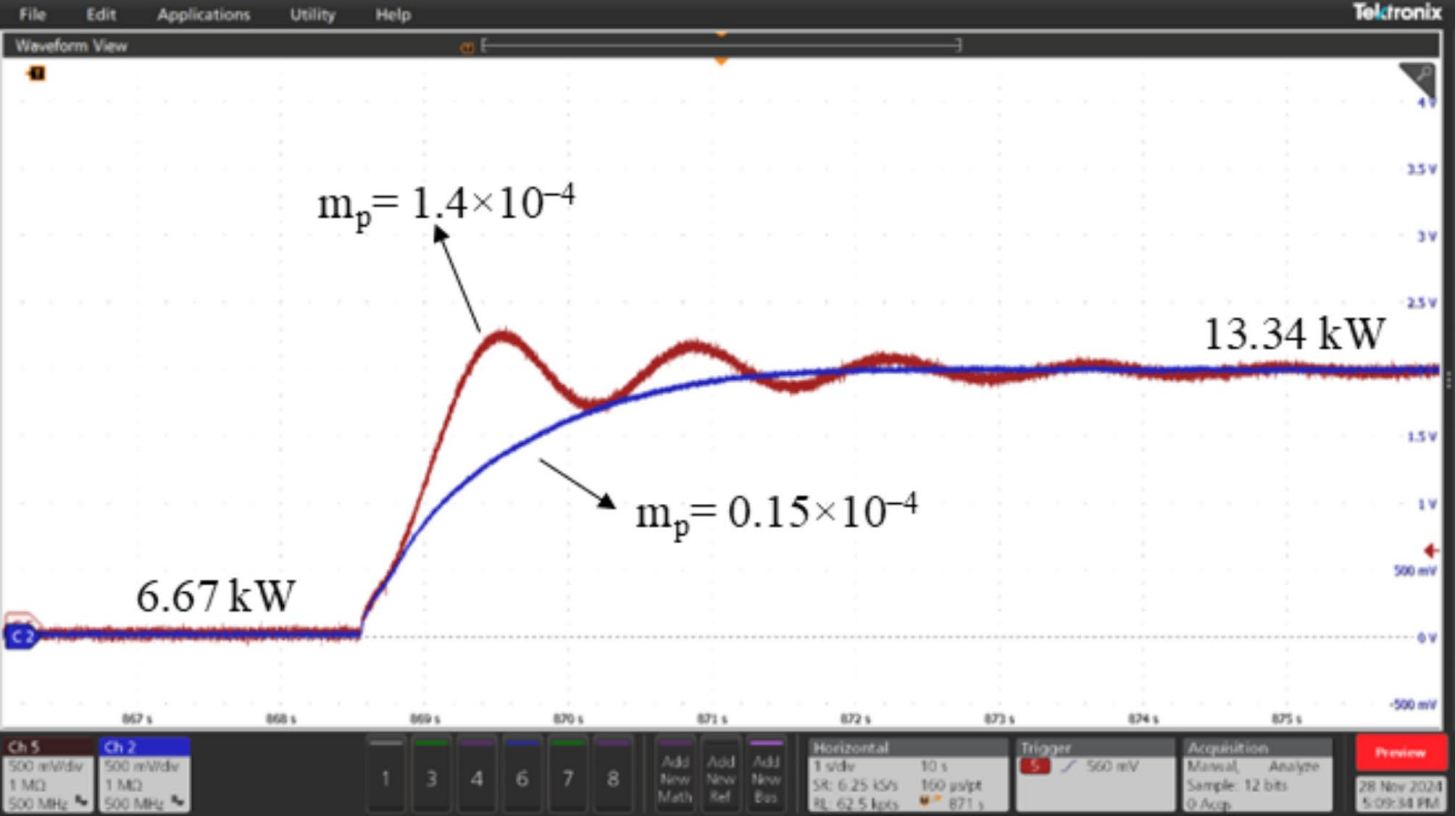
Transient response for DG2 active power at m_p_=0.15 × 10^− 4^ and mp = 1.4 × 10^− 4^ after a step load increase.

**Fig. 15:**
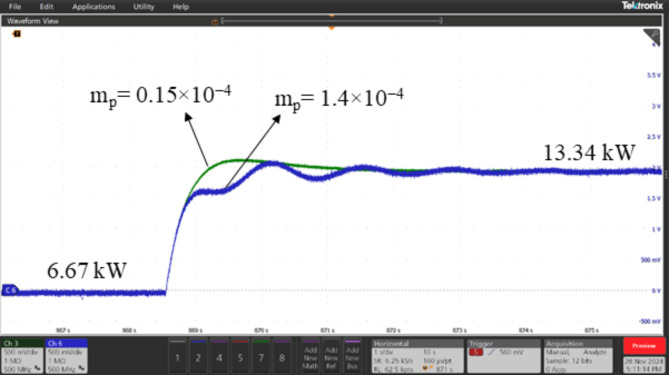
Transient response for DG3 active power at m_p_=0.15 × 10^− 4^ and mp = 1.4 × 10^− 4^ after a step load increase.

The performance of the proposed algorithm was validated through a controller-in-the-loop (CIL) experiment using OPAL-RT real-time simulator. The experimental setup, depicted in Fig. 12, consists of the following components: (1) The OPAL-RT OP5707XG platform for real-time simulation (2) A digital oscilloscope (MSO58B Tektronix) (3) A PC equipped with an Intel^®^ Xeon^®^ W-2245 CPU @ 3.9 GHz and 128 GB of RAM, running OPAL-RT software. The proposed algorithm was tested on the 3-bus network depicted in Fig. 3, comprising three identical DG units, each rated at 15 kVA, supplying a total load of 20 kW and 4 kVAR. A step load increase was introduced using a static RL load of 20 kW and 6 kVAR, and the transient responses of the DGs’ active powers were analyzed for two values of active power droop gain: m_p_=0.15 × 10^− 4^ and m_p_=1.4 × 10^− 4^. It is well-known that increasing the active power droop gain reduces the stability margin, leading to a more oscillatory response following dynamic disturbances in the system. This behavior is evident and can be observed in Figs. 13, 14 and 15, which compare the transient responses of active power after the step load rise for the two specified m_p_ values. After the load disturbance, the dominant modes of the system were estimated using ESPRIT algorithm for both m_p_ values, yielding − 15.276 ± 9.9136i for m_p_=0.15 × 10^− 4^ and − 2.8071 ± 55.895i for m_p_=1.4 × 10^− 4^. These estimations align closely with the transient responses and their oscillatory behavior observed in the OPAL-RT simulations which validate the accuracy and credibility of the proposed algorithm.

## Conclusion

This paper proposes a real-time stability assessment tool based on Kurtosis-ESPRIT algorithm operating on the measurements of intentionally perturbed active power. The Kurtosis measure is uniquely employed for describing the signal’s characteristics used in the identification process of the system’s dominant modes. The proposed stability assessment tool managed to achieve minimal estimation error within all case studies while using local measurements only. When compared to the common Prony, Matrix Pencil, and SID techniques, the proposed algorithm showed superior performance in terms of estimation accuracy. ESPRIT algorithm proved its reliability and accuracy in estimating the most dominant modes in different microgrid benchmarks at various active power droop gains, different variations of line parameters, several levels of noise, and under large load changes and topological disturbances. Additionally, the performance of the proposed algorithm was experimentally validated using OPAL-RT real-time simulator. Thus, the developed algorithm can perform as a robust real-time stability assessment tool that can guide network operators to take corrective actions to ensure microgrids’ stability at different dynamic operating conditions.

## Data Availability

The datasets used and/or analysed during the current study available from the corresponding author on reasonable request.
